# Influence of hyperproteinemia on reproductive development in an invertebrate model

**DOI:** 10.7150/ijbs.33310

**Published:** 2019-08-19

**Authors:** Yong-Feng Wang, Xue-Dong Chen, Guang Wang, Qiu-Ying Li, Xin-Yin Liang, Yang-Hu Sima, Shi-Qing Xu

**Affiliations:** 1 School of Biology and Basic Medical Sciences, Medical College, Soochow University, Suzhou 215123, China.; 2 Institute of Agricultural Biotechnology & Ecology (IABE), Soochow University, Suzhou 215123, China.

**Keywords:** hyperproteinemia, animal model, reproductive development, vitellogenin, programmed cell death

## Abstract

Hyperproteinemia is a severe metabolic disease characterized by abnormally elevated plasma protein concentrations (PPC). However, there is currently no reliable animal model for PPC, and the pathological mechanism of hyperproteinemia thus remains unclear. In this study, we evaluated the effects of hyperproteinemia on reproductive development in an invertebrate silkworm model with a controllable PPC and no primary disease effects. High PPC inhibited the synthesis of vitellogenin and 30K protein essential for female ovarian development in the fat body of metabolic tissues, and inhibited their transport through the hemolymph to the ovary. High PPC also induced programmed cell death in testis and ovary cells, slowed the development of germ cells, and significantly reduced the reproductive coefficient. Furthermore, the intensities and mechanisms of high-PPC-induced reproductive toxicity differed between sexes in this silkworm model.

## Introduction

Abnormal plasma protein concentration (PPC) is closely related to clinical mortality 1-4. Hyperproteinemia is a major metabolic disease characterized by abnormally elevated PPC 5-8, and is clinically common in complications of multiple myeloma 9, renal failure 10, liver disease 11-13, and nematode infections 14. Hyperproteinemia has also been observed in *Leishmania infantum* infections 15 and chronic lymph flow 16 in dogs, peritonitis in cats 17, and abdominal abscesses in horses 18.

There have been few reports of the pathological mechanisms and treatments for hyperproteinemia to date 19,20. However, studies of the relationship between hypoalbuminemia and the physical and chemical properties of blood showed that hyperproteinemia caused blood changes similar to hyperglycemia and hyperlipidemia, leading to increased blood viscosity and to blood circulation and microcirculatory disorders 6,7,21.

Metabolic abnormalities of the circulatory system have been reported to have significant adverse effects on reproductive development. Hypercholesterolemia directly affects fertilization by inducing germ cell apoptosis, worsening Leydig and Sertoli cell secretory functions, reducing sperm production and increasing sperm morphological abnormalities in rabbits 22-24. Hypercholesterolemia and hyperlipidemia during pregnancy inhibited blastocyst development and altered oocyte metabolism, leading to defects in fetal glucose supply and affecting fetal vascular development 25-27. Hyperglycemia in pregnant females may inhibit blastocyst development and affect oocyte metabolism via the hexosamine biosynthetic pathway, causing abnormal oocyte and embryo development 25, subsequently increasing perinatal risk 28. Hyperglycemia has also been shown to damage DNA in germ cells, induce oxidative stress, cause testicular structural changes, and affect male reproductive capacity 29,30. These results highlight the need to evaluate the effects of hyperproteinemia on reproductive development.

There is currently no reliable animal disease model or modeling method for hyperproteinemia in mammals, and we therefore used an invertebrate silkworm model 31-33. We created an animal model of hyperproteinemia (AM) with no primary disease, in which hyperproteinemia could be generated by controlling PPC levels. We then used this model to investigate the effect of hyperproteinemia on the regeneration of fat bodies in metabolic tissues 19. Furthermore, because the circulatory system in silkworms is an open system, organs and tissues, including the gonads, are completely immersed in hemolymph, allowing the direct impact of high PPC on the gonads to be observed. In this study, we reconstructed the silkworm model of hyperproteinemia and evaluated the effect of high PPC on reproductive development and the mechanisms responsible.

## Materials and methods

### Preparation of animals

We used the Dazao strain of *Bombyx mori* in the current study. The larvae were reared conventionally to mature larvae (wandering stage). The spinnerets were then covered with low-melting-point paraffin wax to create a hyperproteinemia model (AM), while silkworms in the control group (CK) were untreated. Both groups were maintained under the same conditions. The experimental process was performed as described previously 19.

### Reproduction testing

Mild hyperproteinemia (mAM) was induced by blocking the silk glands at 6 h after the wandering stage, thus causing approximately 75% of the silk protein to be retained in the silk gland. We then investigated the effect of high PPC on the general fecundity of mated pairs (n=5 control females and n=11 with mild hyperproteinemia), based on oviposition performance in females (total eggs laid per female, including unfertilized and fertilized eggs), and male fertility (rate of fertilized eggs).

### Investigation of gonadal development

Testes (n=3 animals) and ovaries (n=3 animals) were removed from silkworms at the indicated times after modeling, placed in DEPC water, soaked in 4% paraformaldehyde and used to make paraffin sections. The samples were dewaxed in xylene (10 min, twice), followed by 100%, 90%, 80%, and 70% ethanol, and ultrapure water for 10 min each, at room temperature. Sections were stained with hematoxylin-eosin (HE) and used to evaluate gonad development. Silkworm body weights and gonad weights (n=6 animals) were recorded and the gonad somatic index (GSI = weight of gonad/body weight) was determined at 48, 96, and 192 h after modeling.

### Spermateleosis

Spermateleosis was affected by the duration of exposure to high PPC *in vivo* and the duration of culture without high PPC *in vitro*. The animals (n=6 animals) were treated as described previously 34. Testes were isolated using surgical forceps at 48, 96, or 192 h after modeling, and washed three times with pre-cooled physiological saline and culture medium to eliminate adherent hemocytes. The spermatocysts were then released from each testis into the culture medium using forceps, transferred to a 24-well plate, and cultured for 72 h at 25°C without high PPC in Grace's insect cell culture medium supplemented with 10% silkworm hemolymph and appropriate concentrations of antibiotics 34. The numbers and morphology of the developing spermatocysts were then observed.

### Western blotting

Proteins were extracted from Bombyx (n=3 animals) fat bodies in RIPA buffer at 48, 96, and 192 h after modeling. The total protein concentration was determined using a BCA Protein Assay Kit (Solarbio, Beijing, China), and 100 µg of protein extracts were subjected to 10% sodium dodecyl sulfate-polyacrylamide gel electrophoresis and transferred to a polyvinylidene difluoride membrane using a semi-dry transfer film. The membrane was blocked with a blocking solution, followed by incubation with purified anti-vitellogenin (Vg), anti-30Kc19 (1:1000), or anti-tubulin (1:5000) antibody (Cell Signaling Technology, Beverly, MA, USA), and then washed and incubated with horseradish peroxidase (HRP)-labeled anti-rabbit IgG (Bioworld Technology, Minneapolis, MN, USA). The membranes were then photographed using an EZ-ECL chemiluminometer Detection Kit for HRP (Biological Industries, Kibbutz Beit-Haemek, Israel) after 1 min in the dark at room temperature. The resulting images were processed and analyzed.

### Real-time polymerase chain reaction analysis

Gonads, hemolymph, and fat bodies were removed from the silkworms at the indicated time after modeling. Each tissue sample was mixed from three individual silkworms. Transcript levels of Vg (BmVg), 30K proteins (Bm30Kc19), 20-hydroxyecdysone receptor (BmEcR), Vg receptor (BmVgR), early transcription factor 74a (BmE74A), autophagy-related genes (BmAtg6 and BmAtg8), and an apoptosis initiation-related gene (BmDronc) were analyzed by quantitative real-time polymerase chain reaction (qRT-PCR). α-Tubulin was used as a reference gene. qRT-PCR was performed in a 20-μl reaction solution using an ABI StepOnePlus™ real-time PCR system (Ambion, Foster City, CA, USA). Total RNA was extracted by traditional methods for qRT-PCR, and qRT-PCR was performed as described previously 19 using the primers listed in Table S1.

### Gonad staining

Reactive oxygen species (ROS) were stained using a ROS Assay Kit (Solarbio, Beijing, China), as described previously (Yan et al., 2016). Silkworm (n=3 animals) gonads were isolated at the appointed time and collected in normal saline (0.7% NaCl) to avoid exposure to air, quickly placed into ROS-staining solution for 15 min at 37℃, and then washed three times with saline in the dark. After washing, the gonads were placed on a slide (repeated in triplicate) and green fluorescence from the ROS was observed under a fluorescence microscope (Olympus BX51, Tokyo, Japan).

Gonad sections were dewaxed and used for terminal deoxynucleotidyl transferase dUTP nick end labeling (TUNEL) and monodansylcadaverine (MDC) staining using an In Situ Cell Death Detection Kit (TMR Red, Roche, Indianapolis, IN, USA) and MDC staining Kit (Solarbio, Beijing, China), respectively according to their manufacturers' instructions. TUNEL and MDC staining solutions were added to the biopsy tissues (repeated in three tissue sections) for 1 h at 37℃ in the dark and the tissue sections were then washed three times in phosphate-buffered saline for 5 min each. Red fluorescence from the TUNEL assay and green fluorescence from the MDC assay were observed under an Olympus BX51 fluorescence microscope.

## Results

### Impact of hyperproteinemia on reproductive development in silkworms

We previously showed that silkworm larvae with hyperproteinemia (AM) underwent slower metamorphosis after the wandering stage, with no individuals completing metamorphosis and all dying before eclosion 19. We therefore modified this previous method to allow us to obtain adult silkworms and to investigate the impact of high PPC on their reproductive development, using animals with mild hyperproteinemia (mAM). The PPC level was restored in mAM after 192h, but was increased rapidly in AM, while the PPC levels in both groups AM and mAM are increased steadily before 192h (Fig.S1). Although the rate of metamorphosis in mAM silkworms was still reduced, 60% of individuals developed into pupae at 72 h after modeling, and 20% developed into adults at 312 h after modeling.

**Figure 1:**
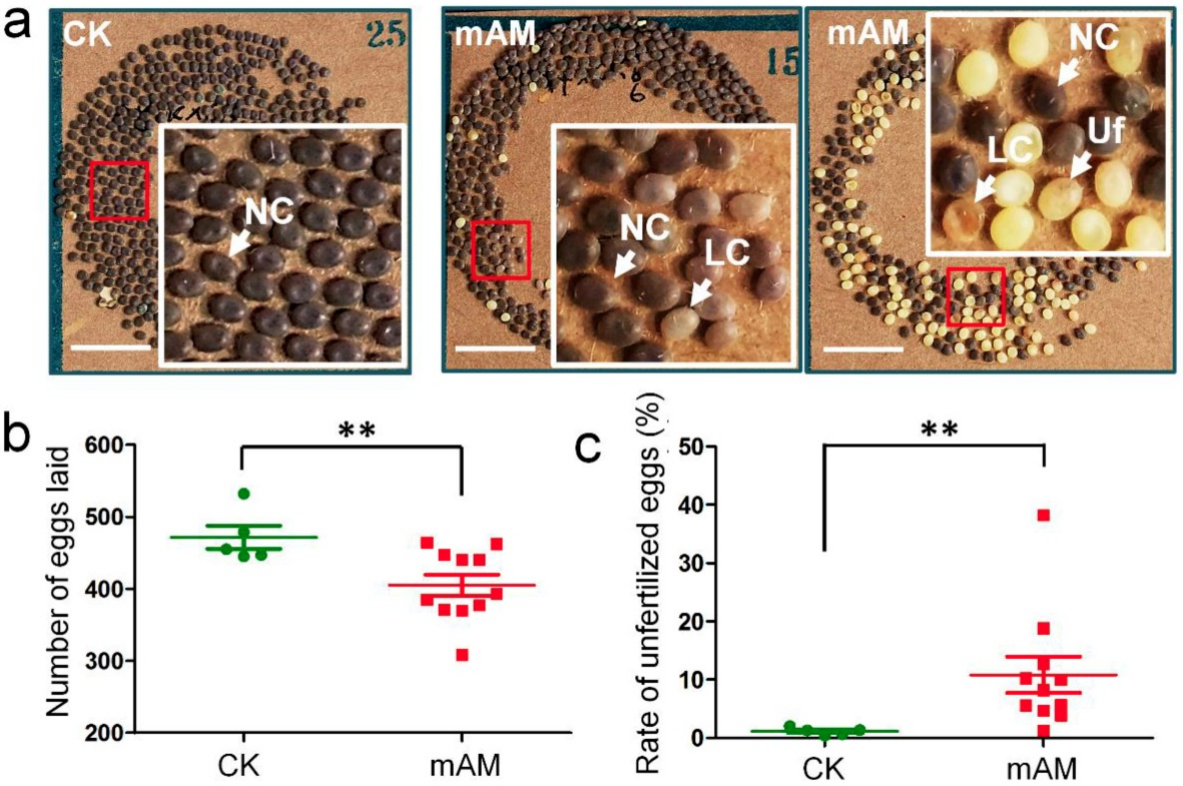
** Eggs laid by adult female silkworms. (a)** Morphologies of eggs laid by control (CK) females and by females with mild hyperproteinemia (mAM). Mild hyperproteinemia was induced 6 h after the wandering stage by blocking the spinnerets, resulting in retention of approximately 75% of the silk protein in the silk gland. NC, natural color eggs; LC, light color of abnormally developed eggs; Uf, unfertilized eggs. **(b)** Number of eggs laid. **(c)** Rate of unfertilized eggs. *P<0.05; **P<0.01 between control and mAM groups (n=5 female adults in control and n=11 in mAM). Bar = 1 cm.

We investigated the fertilization and development of eggs laid by adult mAM silkworms. Eggs in the control group developed fully and showed consistent size and color (dark brown), with very few unfertilized yellow eggs. In contrast, there were many unfertilized yellow eggs and abnormal light-colored eggs in the mAM group (Fig. 1a). The number of eggs laid by females in the mAM group was 14.1% lower than in the CK group (Fig. 1b), and the rate of unfertilized eggs was increased from (1.1 ± 0.65)% in the CK group to (10.7 ± 7.2)% in the mAM group (Fig. 1c). These results indicated that high PPC had significant adverse effects on egg quantity and quality in silkworms, suggesting that hyperproteinemia at the stage of germ cell development adversely affected adult reproduction in silkworms.

To study the mechanism whereby high PPC affected oviposition and fertilization, we reconstructed an animal model of hyperproteinemia (AM) as described by Chen et al. (2018) 19 and investigated the effect of hyperproteinemia on gonad development.

**Figure 2:**
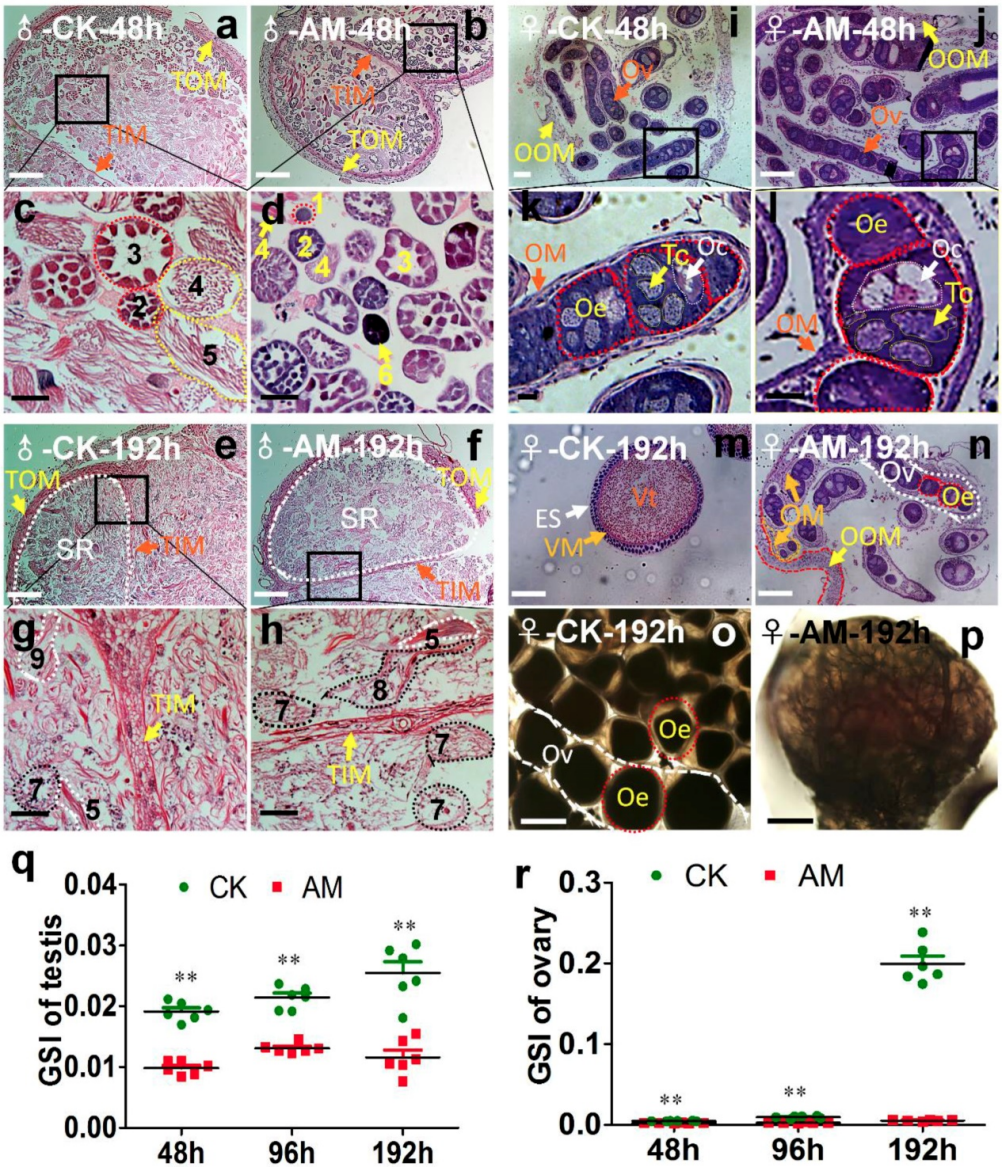
** Impact of hyperproteinemia on gonad development. (a-n)** Sections of gonads stained by hematoxylin-eosin at 48 and 192 h after modeling. **(o, p)** Ovaries under cover glass at 192 h after modeling. TOM, outer testis membrane; TIM, inner testis membrane; SR, spermatogenic room. Numbers 1-3 indicate early developmental stage spermatocysts during the 32-, 64-, and 128-primary spermatocyte stages, respectively; 4 and 5 indicate late developmental stage spermatocysts during spermiogenesis and spermatodesm, respectively; 9 indicates the release of sperm from mature spermatocysts; 6 indicates cacoplastic spermatocysts during early developmental stage; and 7 and 8 indicate cacoplastic spermatocysts during spermiogenesis and cacoplastic spermatodesm, respectively. Ov, ovarioles; OOM, ovarian outer membrane; OM, ovariole membrane; Oe, ooecium; Oc, oocyte; Tc, trophocyte; Vt, vitellin; VM, vitelline membrane; ES, egg shell. (q and r) Gonadosomatic index (GSI) was determined at 48, 96, and 192 h after modeling. **P<0.01 between the AM and control groups (n=6 repeated animals).

Silkworm testes include four spermatogenic rooms filled with developing spermatocysts 34. At 48 h after modeling, we observed a few early developmental stage spermatocysts and numerous late developmental stage spermatocysts in the spermatogenic rooms in CK silkworms during spermiogenesis and spermatodesm (Fig. 2a, 2c). In contrast, we observed numerous early developmental stage spermatocysts in the spermatogenic rooms, but few late developmental stage spermatocysts in the center of the spermatogenic rooms in the AM group, as well as some dysplastic spermatocysts with tightly aggregated nuclei (Fig. 2b, 2d). At 192 h after modeling, the spermatogenic rooms in CK silkworms were filled with sperm released from mature spermatocysts, and very few late developmental stage spermatocysts undergoing spermiogenesis and spermatodesm were observed (Fig. 2e, 2g). In contrast, the spermatogenic rooms in AM silkworms were filled with cacoplastic late developmental stage spermatocysts (Fig. 2f, 2h). Although there was no significant difference in the morphology or weight of the testes between the AM and CK group at 192 h after modeling (Fig. S2b), but the comparable GSI was significantly lower in the AM compared with the CK group at 48, 96, and 192 h (Fig. 2o). These results indicate that hyperproteinemia affected the development of the testis and inhibited sperm formation.

After the oogonia proliferated into primary oocytes in the oviduct in CK silkworm ovaries, each primary oocyte proliferated into eight cells, forming an egg chamber with an oocyte and seven nurse cells. The oocyte absorbed nutrients from the trophoblast and grew further to produce a mature egg 35,36. Ovary weights in female AM silkworms at 48 h after modeling (Fig. S2b) were lower than in the CK group, and the development of the germ cells in ovarian tissue was slower than in the CK group, as shown by HE staining (Fig. 2i, 2j, 2k, 2l). The GSI was significantly lower in the AM group at 48-192 h after modeling compared with the CK group (Fig. 2o). At 192 h, the ovary was fully developed in the CK group and the egg tube was filled with formed eggs, indicating a mature adult ovary (Fig. 2m, 2o). In contrast, eggs in the AM group remained at the same developmental level as at 48 h after modeling (Fig. 2n, 2p). These results showed that ovary growth was slower in AM than in CK silkworms, indicating that hyperproteinemia inhibited ovary development and the formation of eggs in silkworms.

**Figure 3:**
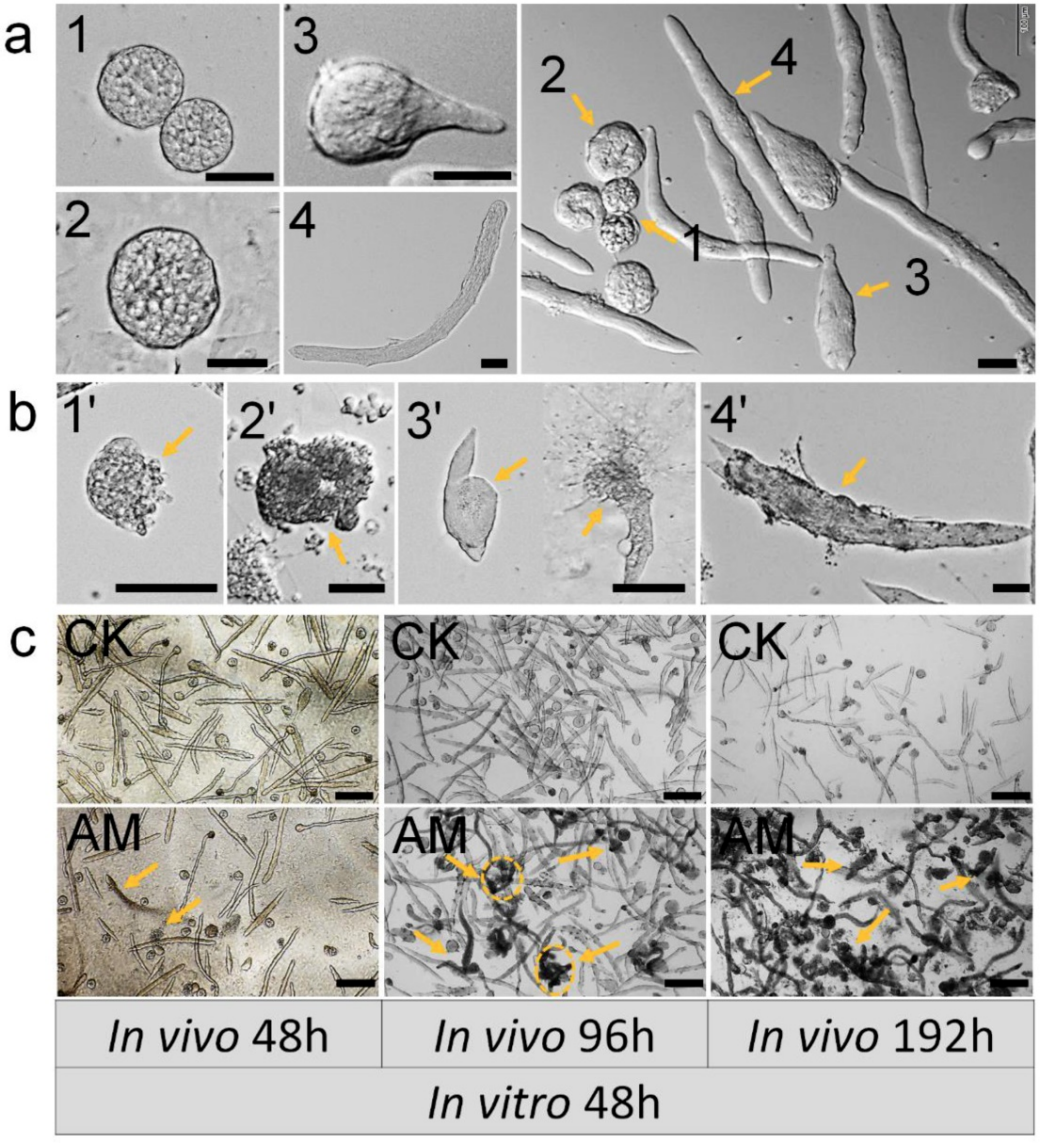
** Impact of hyperproteinemia on spermateleosis in *Bombyx mori*. (a)** Natural morphology of spermatocysts. Numbers 1 and 2 indicate 64-, and 258-stage primary spermatocytes in spermatocyst, respectively; 3 indicates spermatocysts during spermiogenesis; and 4 indicates spermatodesm. **(b)** Cacoplastic morphology of spermatocysts characterized by apoptosis, malformation, and damaged membrane. Numbers 6-9 indicate cacoplastic spermatocysts corresponding to 1-4 in (a). **(c)** Spermateleosis was affected by the duration of high-PPC exposure *in vivo* (i.e., time between model establishment in silkworm larvae and removal of testes). Arrows indicate dysplastic seminal vesicles. Testis were removed at 48, 96, and 192 h after modeling for *in vitro* culture for 48 h. Bars = 50 μm in (a) and (b) and 200 μm in (c).

We assessed the impact of high PPC exposure on male germ cell development quantitatively by *in vitro* culture of spermatocysts, as described previously 34. Normal seminal vesicles can undergo the whole sperm development process from spermatogonia to mature sperm *in vitro*, namely by completing the three developmental stages: primary spermatocytes, spermatocysts during spermiogenesis, and spermatodesm (Fig. 3a).

Testes were removed from male silkworms at 48, 96, and 192 h after hyperproteinemia modeling and cultured *in vitro* for 192 h. Seminal vesicle growth and development were adversely affected as indicated by increased apoptosis, deformity, and membrane damage. Typical dysplastic spermatocysts were seen during the three developmental stages from primary spermatocytes to mature sperm: primary spermatocytes, spermatocysts during spermiogenesis, spermatodesm (Fig. 3b).

Testes were also removed from male silkworms at 48, 96, and 192 h after hyperproteinemia modeling, for *in vitro* culture of spermatocysts for 48 h. Seminal vesicles in the CK group developed normally with the formation of mature sperm bundles, and seminal vesicles with typical dysplasia were rare (Fig. 3c). There was no evidence of dysplastic spermatogenesis in the AM group at 48 h after modeling, but dysplastic and necrotic seminal vesicles were clearly observed after 48 h of *in vitro* culture. Furthermore, AM testes removed at 192 h after modeling showed many spermatozoa with dysplasia, atrophy, and necrosis, and subsequent culture *in vitro* for 48 h resulted in the death of most spermatocysts (Fig. 3c and Fig. S3).

Dysplastic and necrotic seminal vesicles increased in line with the duration of *in vitro* culture in the AM group, irrespective of when the testes were removed (Fig. S3). These results indicated that exposure of male gonads to a high-protein hemolymph environment had significant adverse effects on the development of the seminal vesicles, even after they were removed from the high PPC environment, indicating cumulative and delayed toxicity in silkworm gonads.

### Hyperproteinemia impacts synthesis and transport of female vitellogenin

**Figure 4:**
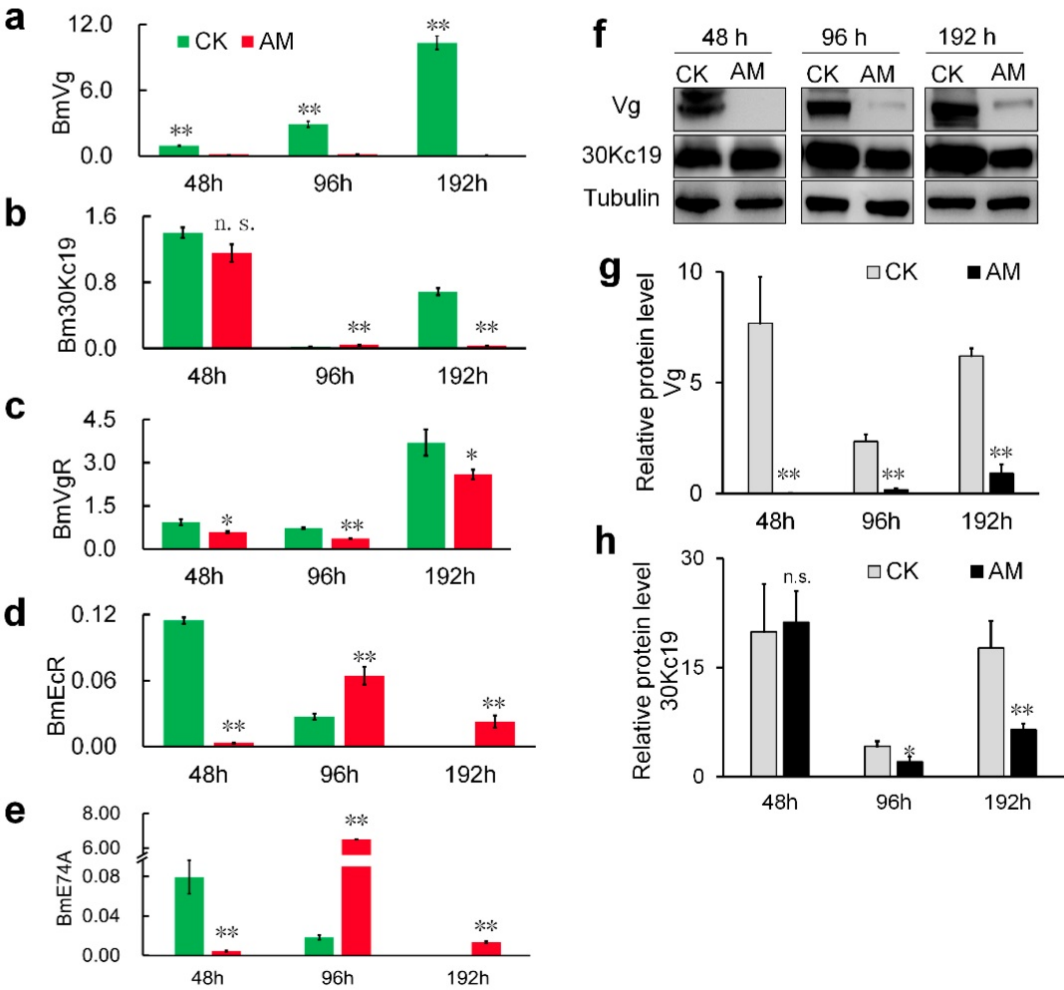
** mRNA levels of (a)** BmVg and **(b)** Bm30Kc19 genes in fat body, (c) VgR gene in ovary, and **(d)** EcR and **(e)** E74A genes in hemolymph. **(f-h)** Vg and 30K proteins in fat bodies detected by western blotting. Fat bodies and ovaries were removed from female silkworms at 48, 96, and 192 h after modeling. *P<0.05; **P<0.01; n.s., no significant difference between the two groups.

We investigated the mechanism whereby high PPC hindered the formation of silkworm eggs by assessing changes in the expression of genes and proteins involved in vitellogenin (Vg) synthesis 36,37. Vg mRNA levels in the fat body of metabolic tissues continued to increase during metamorphosis (48-192 h) in CK group silkworms, but remained low in the AM group (Fig. 4a). 30Kc19 gene transcription was also significantly down-regulated in the AM compared with CK group (Fig. 4b). Changes in Vg and 30Kc19 protein levels (Fig. 4f, 4g, 4h) determined by western blotting confirmed that Vg protein levels were significantly lower in the AM compared with the CK group, while 30Kc19 protein levels were also significantly down-regulated in the middle and late stages (96 and 192 h) of modeling.

Further investigation revealed that transcription of the Vg receptor (VgR) gene was also significantly down-regulated in the AM silkworm ovary (Fig. 4c). Furthermore, transcription of hydroxyecdysone receptor (EcR) , which regulates the transport of Vg from the synthetic tissue in the fat body to the ovary, and transcription of the downstream effector gene E74, were both significantly down-regulated in the hemolymph at 48 h after modeling, but showed significant compensatory up-regulation at 96 and 192 h after modeling (Fig. 4d, 4e). Moreover, expression levels of EcR and E74, which are related to ovarian development, were significantly up-regulated at 48 and 96 h after modeling, and then significantly down-regulated at 192 h, but with phase delays in AM silkworms (Fig. 4d, 4e).

The above results indicated that high PPC inhibited the synthesis of Vg and the silkworm egg-storage protein 30K in the female fat body, inhibited the expression of ovarian VgR via the endocrine pathway, and reduced the transport and accumulation of proteins required for reproduction in the ovary.

### Hyperproteinemia increases programmed cell death in silkworm gonads

We further investigated the mechanism whereby high PPC affected the reproductive development of silkworms in terms of programmed cell death (PCD).

**Figure 5:**
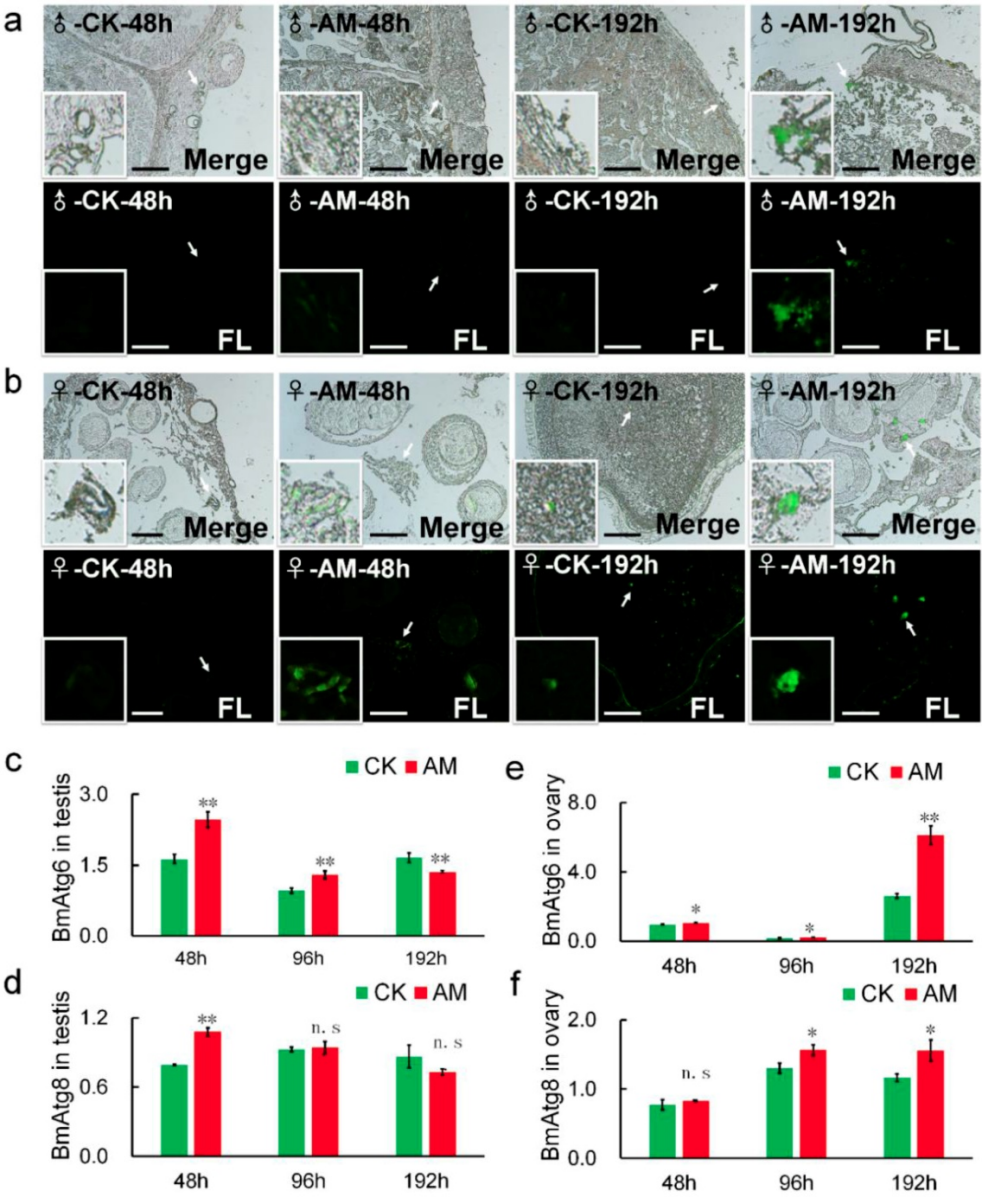
** Changes in the levels of autophagy in testes and ovaries.** Autophagy of silkworm testis **(a)** and ovary **(b)** cells at 48 and 192 h after modeling, demonstrated by MDC staining. **(c-f)** mRNA levels of autophagy-related genes BmAtg6 and BmAtg8 in gonad cells from silkworm at 48-192 h after modeling. *P<0.05; **P<0.01; n.s., no significant difference between the two groups. Bar = 100 μm.

Acidic substances in autophagic vacuole produced by MDC staining is considered a reliable method of assessing the degree of autophagy 38,39,40. Our MDC staining results showed that green fluorescence associated with autophagy were significantly increased in gonad cells from AM silkworms at 48-192 h after modeling, in a time-dependent manner (Fig. 5a, 5b). This suggested that high PPC induced autophagy in silkworm gonad cells. To validate the effects of MDC staining, we investigated the mRNA levels of BmAtg6 and BmAtg8, which are involved in the regulation of autophagy 39,41-43. BmAtg6 and BmAtg8 mRNA levels were significantly up-regulated in AM compared with CK silkworm ovaries, with the change in BmAtg6 occurring before that of BmAtg8 (Fig. 5e, 5f).

Transcription of BmAtg6 and BmAtg8 in testis cells from male silkworms were also significantly up-regulated in the AM group at 48 h after modeling, but down-regulated after 96 h, with the change in BmAtg8 preceding that of BmAtg6 (Fig. 5c, 5d). These results suggested that high PPC may induce autophagy of gonad cells differently in male and female silkworms.

**Figure 6:**
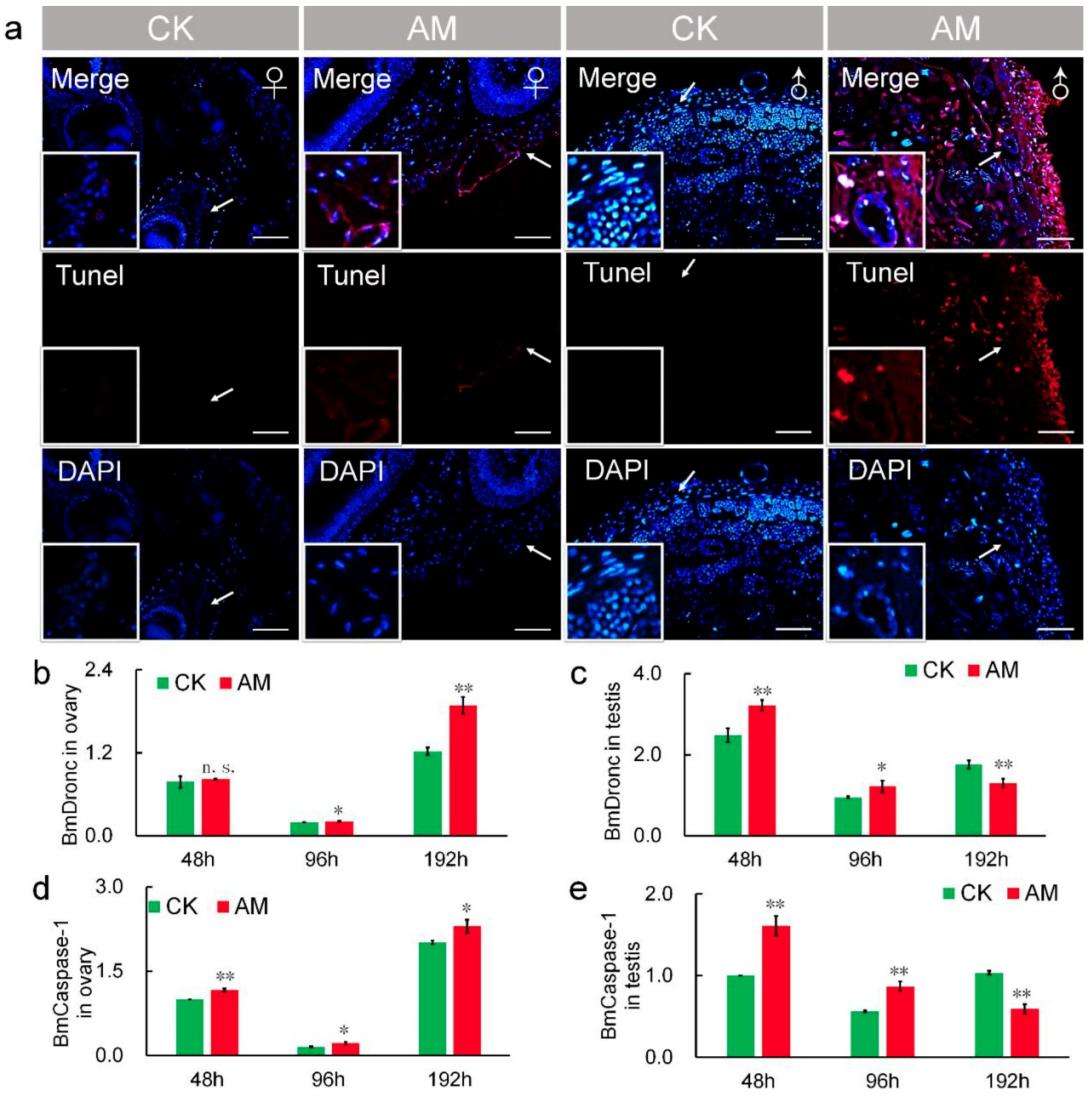
** Hyperproteinemia triggers apoptosis. (a)** TUNEL staining of silkworm gonad sections at 48 h after modeling. **(b-e)** mRNA levels of apoptosis-related genes BmDronc and BmCaspase-1 in gonad cells from silkworm at 48-192 h after modeling. *P<0.05; **P<0.01; n.s., no significant difference between the two groups. Bar = 100 μm. Red represents apoptotic cells.

It has been reported that TUNEL staining is a reliable method of assessing the degree of apoptosis 44,45. Thus, we also investigated the effects of high PPC on apoptosis in gonad cells by TUNEL staining. Apoptosis, indicated by red fluorescence, was detected at 48 h after modeling (Fig. 6a). Apoptosis was increased in AM compared with CK silkworms, and was greater in male compared with female gonads. TUNEL staining was enhanced at 192 h after modeling compared with 48 h regardless of sex, indicating a time-dependent effect of high PPC-induced apoptosis (Fig. S4).

Transcript levels of the apoptosis-initiating gene BmDronc were similar in AM and CK ovaries at 48 h after modeling, but were higher in AM ovaries after 48 h (Fig. 6b). While BmCaspase-1 mRNA levels were significantly up-regulated at 48, 96 and 192 h after modeling (Fig. 6d). BmDronc and BmCaspase-1 mRNA levels in the testis were significantly up-regulated at 48 and 96 h after modeling, but were significantly down-regulated compared with the CK group (Fig. 6c, 6e). These results suggest that high PPC enhanced the apoptosis regulatory signal in gonad cells and further confirmed that high PPC- induced PCD level are different in ovary and testis cells.

**Figure 7:**
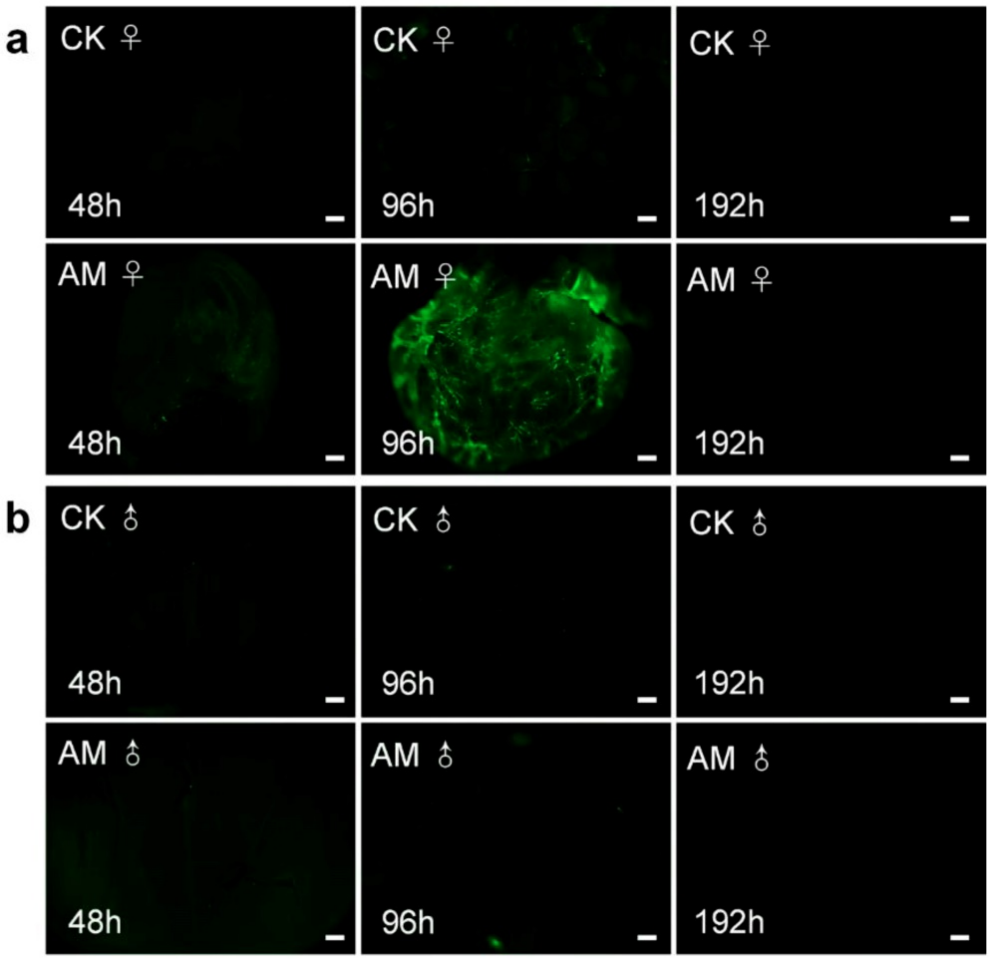
** Impact of hyperproteinemia on reactive oxygen species in silkworm (a)** ovary and **(b)** testis. Bar = 200 μm.

We clarified the role of high PPC in the induction of PCD in silkworm gonad cells by examining ROS levels (Fig. 7). ROS plays an important role in maintaining cell homeostasis^34^, ^46^-^48^. ROS levels in ovary sections (indicated by green fluorescence) were increased after 96 h of modelling compared with the CK group; however, ROS were not clearly observable at other times. ROS levels in male testis were similarly low in CK and AM tissue sections. These results indicate that high PPC induced PCD in silkworm gonad cells, but that this was not completely dependent on ROS, at least in male gonads.

## Discussion

### Sex difference in reproductive toxicity of hyperproteinemia in silkworms

There have been few reports of the effects of hyperproteinemia on the reproductive system of humans and animals to date. In this study, we created a silkworm model of mild hyperproteinemia (mAM) and confirmed that high PPC significantly reduced the adult reproductive coefficient and fertilization rate. We also examined the effect of high PPC on reproductive system development using our earlier hyperproteinemia model (AM), and showed that gonad coefficients were significantly reduced in both female and male silkworms, with reduced rates of germ cell development. Furthermore, male silkworms were more sensitive to PPC than females. These results indicate the further studies are needed to examine the effects of hyperproteinemia on male reproductive toxicity.

**Figure 8:**
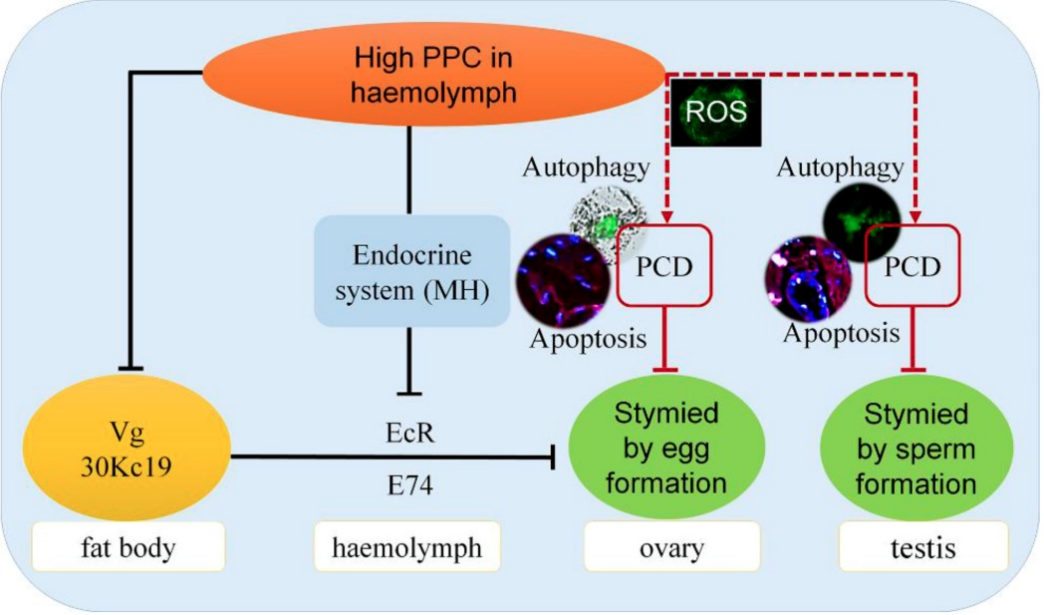
Impacts of hyperproteinemia on gonad development in male and female silkworms.

Numerous studies have confirmed that cells can produce oxidative stress and PCD in response to diseases, including metabolic diseases such as hypertension and hyperglycemia, and malignant tumors such as colorectal cancer 49-52. PCD, including apoptosis and autophagy, plays an important role in maintaining homeostasis 53. In the current study, high PPC induced autophagy and apoptosis in silkworm gonad cells, with differences in both intensity and mechanism between the sexes, including significant differences in ROS levels. Previous studies indicated that the protective function of autophagy was superior to its potential cytotoxic effects 54, and increased levels of autophagy can remove mitochondria and proteins from ROS damage 55,56. In Fig. 5, the expression of BmAtg6 and BmAtg8 were lower in testes of AM at 192h, while the MDC staining revealed that autophagy was significantly upregulated in AM. The results of a similar lack of coordination were found in Fig.6 between BmDronc and BmCaspase-1 mRNA levels and TUNEL staining revealed that apoptosis. These results suggested that testis cells are reduced autophagy/apoptosis signal strength, though cells have been a certain amount of autophagy/apoptosis in AM at 192h. We hypothesized that PPC increased rapidly in female silkworms with hyperproteinemia, and gonad cells maintained homeostasis by initiating autophagy through an increased ROS oxidative stress response and further cell death (Fig. 8).

### Hyperproteinemia affects reproductive development via the endocrine system in silkworms

The main proteins in silkworm eggs include vitellin (Vt, 40%), 30K (35%) and egg-specific protein (25%). Vg is a precursor of Vt, and Vg and 30K are synthesized by fat bodies and secreted into the hemolymph, from where they are absorbed and accumulate in the ovaries 57,58. Germ cells in the ovary have been shown to take up Vg via endocytosis mediated by VgR in the cell membrane in Drosophila melanogaster 59, Aedes aegypti 60, and *B. mori* 61. The ecdysteroid, molting hormone (MH), regulates the uptake of Vt by oocytes through dual control of the EcR in the hemolymph and the early response gene E74 60,62-64, and induces transcription of Vg in female silkworm fat bodies 61. Disruption of EcR function has also been reported to affect the growth of ovaries, oocytes, and eggs 65-67. The Vg protein can play an important role in female reproductive development in B. mori by inhibiting Vg expression and thus adversely affecting egg formation and embryo development 68.

We recently showed that a high PPC environment hindered remodeling of the fat body, as the main metabolic tissue in the silkworm, though this phenotype could be largely rescued by the active ingredient of exogenous MH, 20E 19. In the current study we showed that high PPC inhibited the synthesis of Vg and 30K in female fat bodies, and may further inhibit the expression of VgR in ovarian cells via the MH pathway, thus preventing the transport and accumulation of proteins essential for ovarian development (Fig. 8).

## Conclusion

High PPC inhibits the synthesis of Vg and 30K, which are required for the development of ovaries in female B mori. High PPC is transported through the hemolymph to the gonads and induces PCD in testis and ovary cells, slows the development of germ cells, and significantly reduces the reproductive coefficient. The reproductive toxicities of high PPC differ in terms of intensity and mechanism between the sexes.

## Supplementary Material

Supplementary figures and tables.
