# Supplementary material for: Influence of hyperproteinemia on reproductive development in an invertebrate model
Source: Int J Biol Sci. 2019 Aug 19;15(10):2170–81. doi: 10.7150/ijbs.33310 (PMC6775287; doi:10.7150/ijbs.33310)
Supplement: Supplementary file 1 — Supplementary figures and tables. [file ijbsv15p2170s1.pdf]

Supplemental Materials

Reproductive development influenced by hyperproteinemia in an invertebrate model

Table S1 Primers for qRT-PCR

Genes	Primers	Forward (5′ to 3′)	Reverse (5′ to 3′)
Vitellogenin	BmVg	cttgtgccatcgatagaacag	gtcgatattgcatccccatc
Vitellogenin receptor	BmVgR	gagtgcctgggcgaggatgt	ctgagcgtctggcttgtga
30K proteins	Bm30Kc19	acaggactcgacgctccaaag	gctgctcattatcattggttcg
20-hydroxyecdysone receptor	BmEcR	ccacgatgcctttaccaatg	gtcgaggtgcaggacctttc
Early transcription factor 74a	BmE74A	accgcgttcgacaacttcgat	ctgcaatctttgctgcccgtt
Autophagy-related gene 6	BmAtg6	gttatacggttcgggtgg	tggagtacgcatgtggtg
Autophagy-related gene 8	BmAtg8	aaggctaggcttggagac	cagatgtgggtggaatga
Apoptosis initiation related gene	BmDronc	tgtggctgtcttccttc	atctaagtctgtgccctc
α-Tubulin	Bmα-Tub	ctccctcctccataccct	atcaactaccagccaccc
cysteinyl aspartate-specific proteinases-1	BmCaspase-1	cttcactgctgataaatgtcc	tttctccaagagtaataacctg

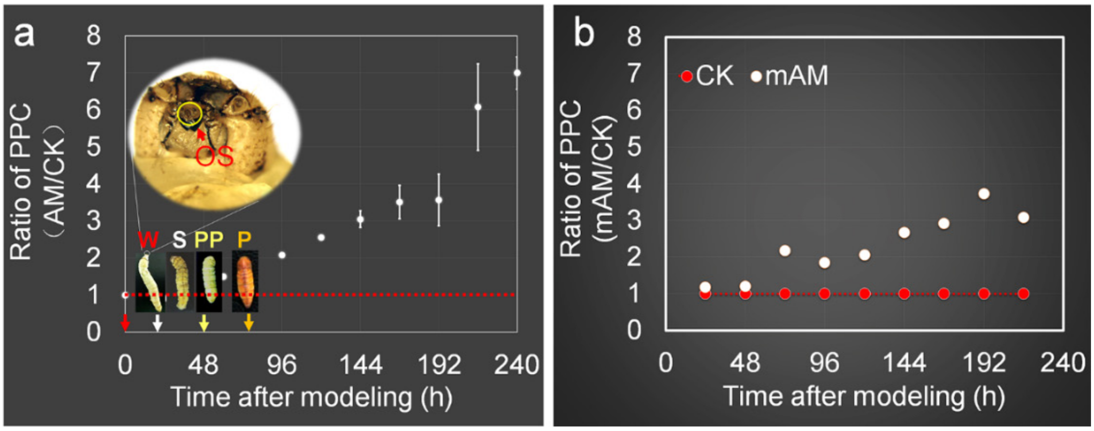

Figure S1 Changes in the plasma protein concentration (PPC) (Figure S1a Data are refer to Chen et al., 2018, and Figure S1b are survey data this work). (a) The ratio of PPC in the animal model (AM) of hyperproteinemia and the CK group. The spinneret of mature *B. mori* larvae in the AM group was covered with low-melting-point paraffin wax at the wandering stage. (b) The ratio of PPC in the moderate animal model (mAM) of hyperproteinemia and the CK group. The spinneret of larvae in the mAM group was covered with low-melting-point paraffin wax at 24h after the wandering stage (that is after spinning 17h spinning and approximate 50% silk protein stuck in silk gland). W, the wandering stage; S, the spinning stage; PP, the pre-pupal stage; P, the pupal stage; A, the adult stage; OS, orifice of the spinneret. * and ** indicate that the differences between the two groups reached significance levels of $P<0.05$ and $P<0.01$, respectively ($n=3$).

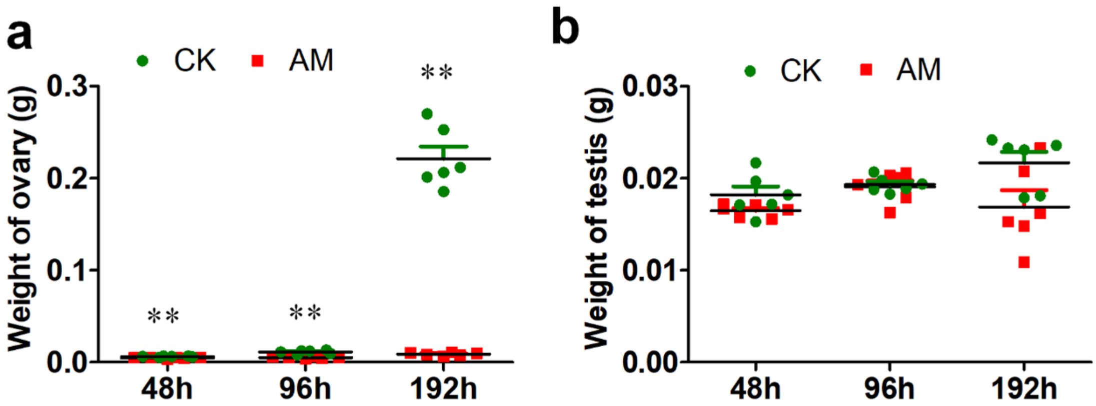

Fig.S2 Weight of gonads are determined at 48h 96h and 192h after modeling. **, the difference between the two groups of AM and CK reached the significant level of P<0.01 (n=6 repeated organisms).

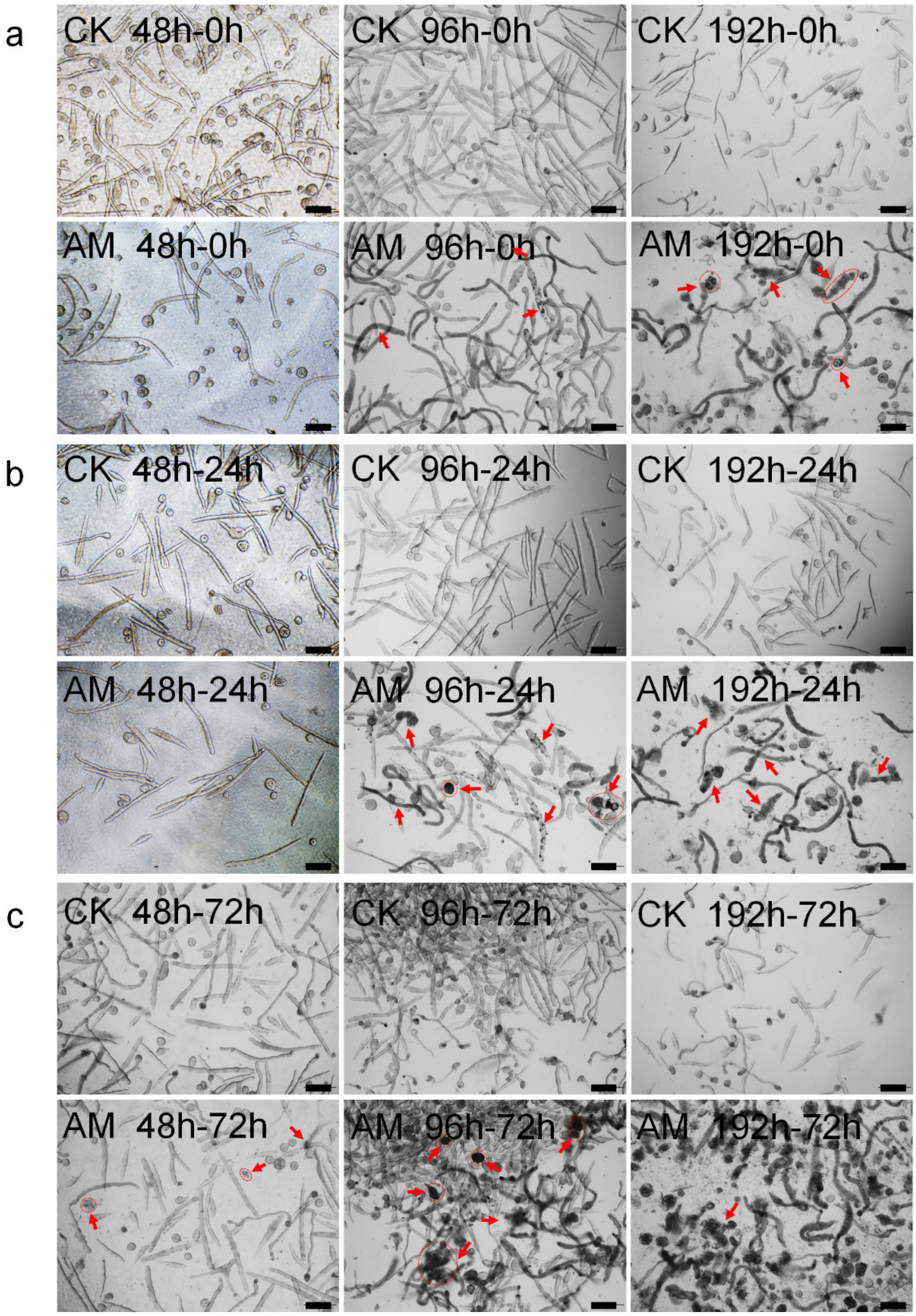
a
CK 48h-0h
CK 96h-0h
CK 192h-0h
AM 48h-0h
AM 96h-0h
AM 192h-0h
b
CK 48h-24h
CK 96h-24h
CK 192h-24h
AM 48h-24h
AM 96h-24h
AM 192h-24h
c
CK 48h-72h
CK 96h-72h
CK 192h-72h
AM 48h-72h
AM 96h-72h
AM 192h-72h

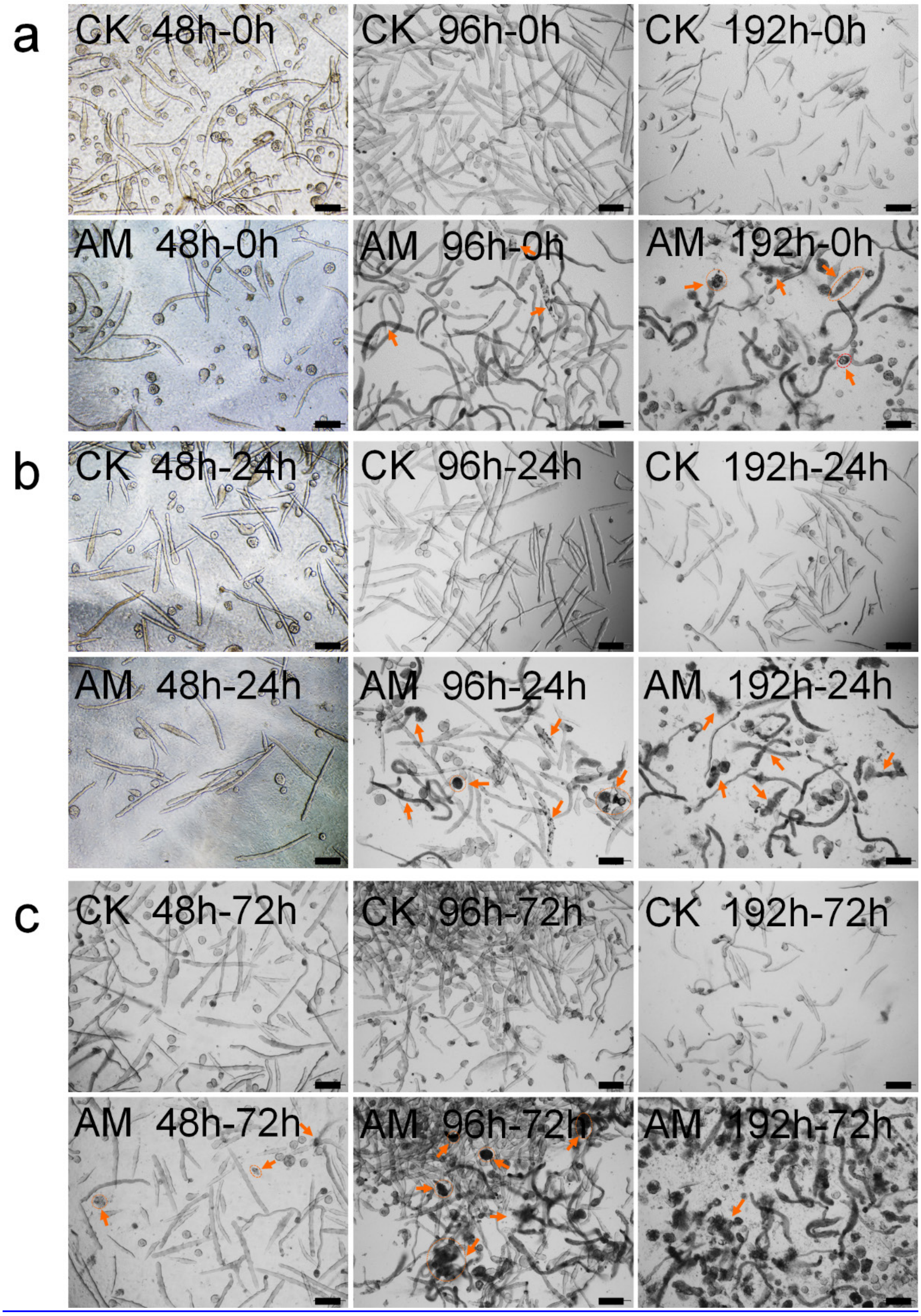

Fig.S3 The impact of hyperproteinemia on spermateleosis of *Bombyx mori*. The method of a joint investigation *in vivo*/*in vitro* was used. (a) 48h-0h, 96h-0h and 192h-0h indicate the spermatocyst removed from larvae after modeling 48h, 96h and 192h, respectively. (b) 48h-24h, 96h-24h and 192h-24h indicate the spermatocyst removed from larvae after modeling 48h, 96h and 192h, respectively. Following cultured *in vitro* 24h. (c) 48h-72h, 96h-72h and 192h-72h indicate the spermatocyst removed from larvae after modeling 48h, 96h and 192h, respectively. Then, cultured *in vitro* 72h. n=3, Bar=200μm.

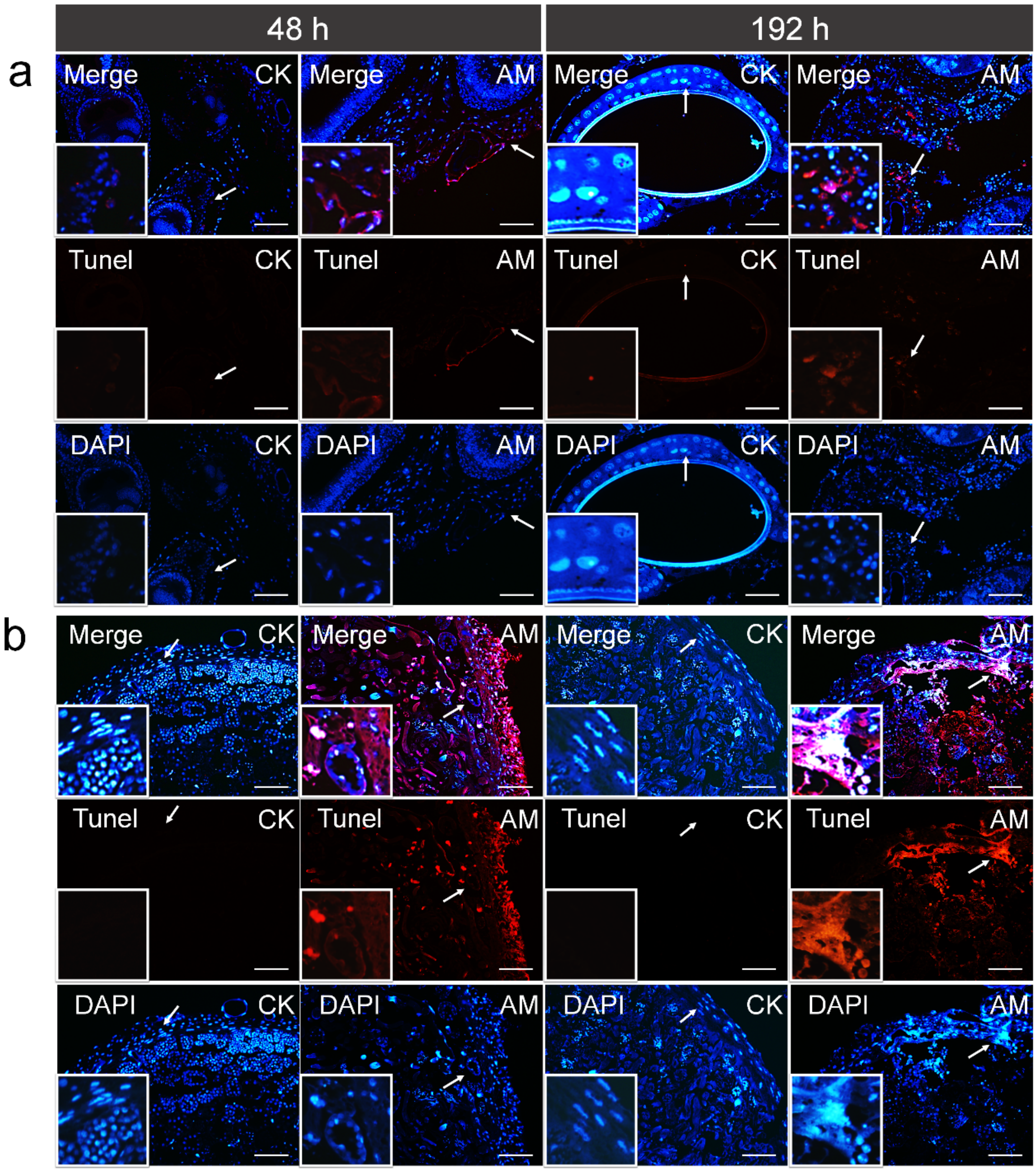

Fig.S4 Hyperproteinemia induces apoptosis. TUNEL staining results of female silkworm gonad sections (a) and male silkworm gonad sections (b) at 48h after modeling. Red represents apoptotic cells. n=3, Bar = 100 um.
